# Anionic polyacrylamide influence on the lead(II) ion accumulation in soil – the study on montmorillonite

**DOI:** 10.1007/s40201-020-00485-w

**Published:** 2020-05-23

**Authors:** G. Fijałkowska, K. Szewczuk-Karpisz, M. Wiśniewska

**Affiliations:** 1 Department of Radiochemistry and Environmental Chemistry, Institute of Chemical Sciences, Faculty of Chemistry, Maria Curie-Sklodowska University in Lublin, M. Curie-Sklodowska Sq. 3, 20-031 Lublin, Poland; 2 Institute of Agrophysics, Polish Academy of Sciences, Doświadczalna 4, 20-290 Lublin, Poland

**Keywords:** Adsorption, Zeta potential, Surface charge density, Heavy metal immobilization, Aggregation

## Abstract

**Purpose:**

Polymeric substances, as soil conditioners, limit the erosion process as well as improve the soil structure. The same macromolecular compounds may influence the heavy metal accumulation in soil environment. The main aim of this study was investigation of anionic polyacrylamide (AN PAM) effect on the lead(II) ion sorption on the montmorillonite surface. The effects of Pb(II) ion concentration, sequence of heavy metal and anionic polymer addition into the system as well as anionic group content in the PAM macromolecules were also studied.

**Materials and methods:**

The study was performed on montmorillonite (clay mineral). Two types of polymers were used: AN PAM 5% and AN PAM 30% containing 5% and 30% of carboxylic groups, respectively. The adsorbed amounts of Pb(II) ions or AN PAM on the solid were determined spectrophotometrically. Electrokinetic properties of the examined systems were established using potentiometric titration and microelectrophoresis method. The montmorillonite aggregation without and with selected substances was described based on the sedimentation study.

**Results:**

At pH 5 the Pb(II) adsorbed amount on montmorillonite equaled 0.05 mg/m^2^ (for the initial concentration 10 ppm). Anionic polyacrylamide increased this value significantly (it was 0.11 mg/m^2^ with AN PAM 5% and 0.11 mg/m^2^ with AN PAM 30%). The lead(II) ions presence causes a slight increase of the anionic PAM adsorption on the montmorillonite surface. For example, for the initial polymer concentration 100 ppm, the AN PAM 5% adsorbed amount without Pb(II) equaled 0.49 mg/m^2^, whereas with Pb(II) – 0.57 mg/m^2^. What is more, anionic polyacrylamide and lead(II) ions affected electrokinetic properties and stability of the montmorillonite suspension.

**Conclusions:**

Anionic polyacrylamide makes the Pb(II) accumulation on the montmorillonite surface larger and, as a consequence, reduces the Pb(II) availability to organisms. Therefore, this macromolecular compound can certainly be used to remediate soils contaminated with heavy metals.

## Introduction

Recently, contamination of water, air and soil with heavy metals is one of the most distressing environmental issue. Due to development of civilization, the number of contaminated places becomes larger and larger. Gas and dust emissions from smelters, chemical industry, sewage and waste; transport emissions; mining and processing industry of non-ferrous ores as well as contaminated mineral fertilizers are the most common anthropogenic sources of heavy metals [1, 2]. The accumulation of toxic elements in the environment depends on many factors, e.g. physicochemical properties of the element, its tendency to form soluble complexes, water solubility as well as reduction/oxidation potentials. The environmental factors, such as pH value and ionic strength, are also important. Usually, heavy metals are immobilized on the soil minerals (due to chemisorption or physical sorption processes) or taken up by the plants. The toxicity of selected compound to organisms is dependent on, inter alia, its solubility in body fluids and lipids, immunity of organism as well as exposure time [3–9].

Lead (Pb) is a heavy metal that is widely used in industry. It gets into the environment through transport and industrial emissions from steel mills, cement plants and steelworks. It is widely distributed in water and soil, where occurs in the form of soluble or sparingly soluble salts [10–14]. At low concentrations, lead favours the nitrification and inhibits ammonification simultaneously. However, its excessive amount has a negative effect on the basic life processes. Lead disturbs photosynthesis, cell division, nitrogen metabolism and water management. It changes protein synthesis and ATP production in human or animal organisms. What is more, lead has strong embryotoxic, carcinogenic and mutagenic effects. It can disrupt neurological, psychological and reproductive functions. The scale of disorders depends mainly on the amount of element introduced into the body [1, 15–22].

Nowadays, many researchers are working on heavy metal removal using specific materials. Adamczuk and Kołodyńska [23] produced sorbents from fly ash (FAI) and used them in zinc(II) removal. Szewczuk-Karpisz et al. [24] studied the adsorption properties of hay-based activated biochars relative to copper(II) ions and ionic polyacrylamide. In turn, Wiśniewska and Nowicki [25] investigated simultaneous removal of lead(II) ions and poly(acrylic acid) using biocarbons obtained from corncon and peanut shell precursors. Removal technologies for the substances other than heavy metals are also being developed. Kalhor et al. [26] described synthesis and adsorption properties of amino functionalized silica nano hollow sphere as an adsorbent of imidacloprid pesticide. Kamaranifar et al. [27] focused on removal of reactive red 195 dye using powder and ash of barberry stem. In turn, Rafati et al. [28–31] studied removal of ibuprofen, phosphate and naproxen using various innovative solids.

The presented paper focused on the lead(II) accumulation on the montmorillonite surface without and with anionic polyacrylamide (soil flocculant/conditioner). The impacts of Pb(II) ion concentration, sequence of heavy metal and polymer addition into the system as well as anionic group content in the PAM macromolecules were studied. Aggregation, adsorption and electrokinetic (surface charge density, zeta potential) properties of the examined systems were determined using spectrophotometry UV/Vis, potentiometric titration, microelectrophoresis method and sedimentation study. The selected polymeric substance is used in agriculture to increase the soil structure stability. It connects loose particles and favours the formation of large aggregates. As a result, the erosion phenomena are limited or inhibited [32–44]. Soil flocculants can also bind chemical compounds present in soil including heavy metals. However, their influence on this process has not yet been fully understood and explored. The adsorption of heavy metals on the clay minerals in the presence of macromolecular compound is rarely describe in the literature. Due to this fact, the presented study is of great importance. It allows to better understanding of the impact of widely used flocculant (AN PAM) on the soil environment condition. Moreover, it indicates whether anionic polyacrylamide may be used in soil remediation as a substance enhancing the Pb(II) immobilization and reducing availability of this heavy metal to plants and animals.

## Experimental

### Materials

**Fig. 1:**
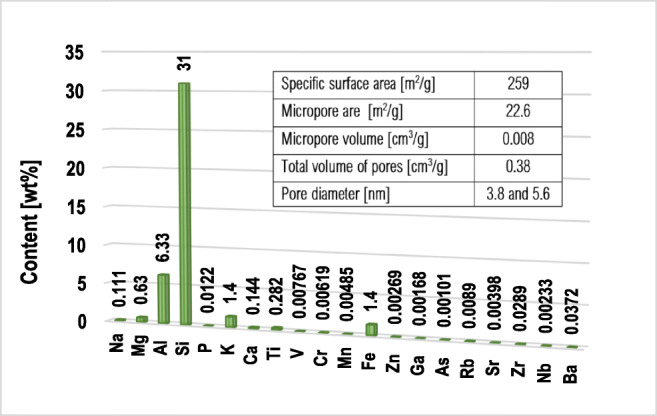
Textural properties and elemental composition of the montmorillonite

Montmorillonite – three-layered aluminosilicate (*Sigma-Aldrich*), was used as an adsorbent in the experiments. Specific surface area and its porosity were determined using the low-temperature nitrogen adsorption/desorption method (*Micromeritics* ASAP 2020 analyzer). The elemental composition of the mineral was determined by the XRF technique (*Panalytical* ED-XRF type Epsilon 3 spectrometer). The obtained results are presented in Fig. 1.

The anionic polyacrylamide (AN PAM) delivered by *Korona* with different anionic group contents i.e. 5% and 30% and molecular weight of 13,000 kDa and 14,000 kDa, respectively, was applied as an adsorbate in the study. The pK_a_ values of these polymers, determined by the potentiometric titration method are 3.3 and 3.2. The dissociation degree (α) of the AN PAM carboxyl groups was also calculated using the following formula [45]:

1 $$ pH-p{K}_a=\mathit{\log}\frac{\propto }{1-\propto } $$

The dissociation degree of AN PAM 5% at pH 3 is 33.4%, at pH 5–98%, at pH 7–99.98% and at pH 9–99.99%. In the case of AN PAM 30%, the α value at pH 3 equals 38.7%, at pH 5–98.7%, at pH 7–99.98%, whereas at pH 9–99.99%.

## Methods

### Adsorbed amount determination by the use of spectrophotometry

All adsorption measurements were conducted at 25 °C and at pH 5 ± 0.1. This pH value was selected because Polish soils are often characterized by such a reaction. Moreover, at pH > 7 lead hydroxide can be precipitated from the aqueous solution. As a supporting electrolyte, 0.001 mol/dm^3^ NaCl was used. The adsorbed amounts of AN PAM and Pb(II) ions on the montmorillonite surface were determined based on decrease in their concentration in the solution after the adsorption process. The anionic polyacrylamide concentration was established using hyamine 1622 [46]. The absorbance of white PAM-hyamine complex was measured spectrophotometrically (spectrophotometer Carry 1000*,* Varian) at the wavelength 500 nm after 15 min of the indicator addition. The concentration of lead(II) was determined based on the reaction of Pb(II) ions with 4-(2-pyridylazo)-rezorcinol (PAR) in the ammonium buffer (pH 10) [47]. The absorbance of red chelate complex (Pb:PAR ratio is 1:1) was determined spectrophotometrically at a wavelength 520 nm. The adsorption process was carried out under continuous shaking (water bath OLS 200, Grant) for 24 h. This time was established based on the previous kinetics study [48]. It ensured equilibrium in the examined systems, even in those complicated containing both heavy metal and polymer. In each examined system the montmorillonite weight was 0.003 g. This weight was optimal (the smaller was impossible to weigh, while the higher one adsorbed all polymer macromolecules from the solution). The appropriate pH value (pH 5) of the examined suspensions were adjusted with a pH-meter (Beckman Instruments). After the adsorption completion, the solids were centrifuged using a microcentrifuge (MPW Med. Instruments) and the clear solutions were collected for further quantitative analysis. The initial AN PAM concentration in adsorption measurements (both adsorbed amount and kinetics) was 100 ppm, whereas the Pb(II) one – 1, 10 and 100 ppm.

### Surface charge density determination by the use of potentiometric titration

The surface charge density and the point of zero charge (pzc) of montmorillonite were determined using the potentiometric titration method. The titrations were conducted in the absence and presence of PAM and/or Pb(II) at 25 °C in the pH range of 3–9 or 3–7. The first pH range was selected for the systems without additives and containing only PAM, whereas the second one – for the systems containing Pb(II) to avoide the lead hydroxide precipitation. The suspensions were prepared using 0.1 g of the mineral and 0.001 mol/dm^3^ NaCl as a supporting electrolyte. The concentrations of AN PAM and Pb(II) ions in the examined systems were equal to 100 ppm and 1 ppm, respectively. The applied set was composed of: thermostated Teflon vessel (thermostat RE 204, Lauda), glass and calomel electrodes (Beckman Instruments), pH-meter PHM 240 (Radiometer) and microburette Dosimat 765 (Methrom) as well as computer. The solid surface charge density (σ_0_) was calculated by special program Titr_v3 (author W. Janusz) using the following equation:

2 $$ {\sigma}_0=\frac{\varDelta V\cdotp c\cdotp F}{m\cdotp S} $$

where: *ΔV* – the difference in the base volume added to the suspension and the supporting electrolyte solution that leads to the specific pH value, *c* – the base concentration, *F* – the Faraday constant, *m* – the mineral mass in the suspension, *S* – the solid surface area.

### Zeta potential determination by the use of Doppler laser electrophoresis

Similar to potentiometric titration, zeta potential of montmorillonite without and with AN PAM and/or Pb(II) ions was determined at 25 °C in the pH range of 3–9 or 3–7. The montmorillonite suspensions were prepared using 0.1 g of the mineral and 0.001 mol/dm^3^ NaCl as a supporting electrolyte. Each suspension was sonicated for 3 min (ultrasonicator XL 2020, Misonix) and divided into 7 parts (in each of them different pH value was adjusted). The concentrations of AN PAM and Pb(II) ions were equal to 100 ppm and 1 ppm, respectively. The electrophoretic mobility (*U*_*e*_) of the particles was measured using a zetasizer Nano ZS (Malvern Instruments) and universal dip cell [49]. Zeta potential (ζ) value was calculated using the Henry equation [50]:

3 $$ {U}_e=\frac{2{\varepsilon}_0\varepsilon \zeta}{3\eta }f\left(\kappa a\right) $$

where: *ε* – the dielectric constant, *ε*_*0*_ – the electric permeability of vacuum, *η* – the viscosity of liquid medium, *f(κa)* – the Henry function.

### Suspension stability determination by the use of spectrophotometry

The changes in the montmorillonite suspension stability/turbidity without and with AN PAM and/or Pb(II) ions were monitored using the sedimentation method. The absorbance was measured in the function of time at the wavelength 500 nm (spectrophotometer Carry 1000*,* Varian). The total time of experiment was 3 h. The concentrations of AN PAM and Pb(II) ions in the studied suspensions were equal to 100 ppm and 1 ppm, respectively. The suspension was prepared using 0.1 g of the solid and 0.001 mol/dm^3^ NaCl as a supporting electrolyte.

## Results and discussion

### AN PAM and Pb(II) ions adsorbed amounts on the montmorillonite surface

**Fig. 2:**
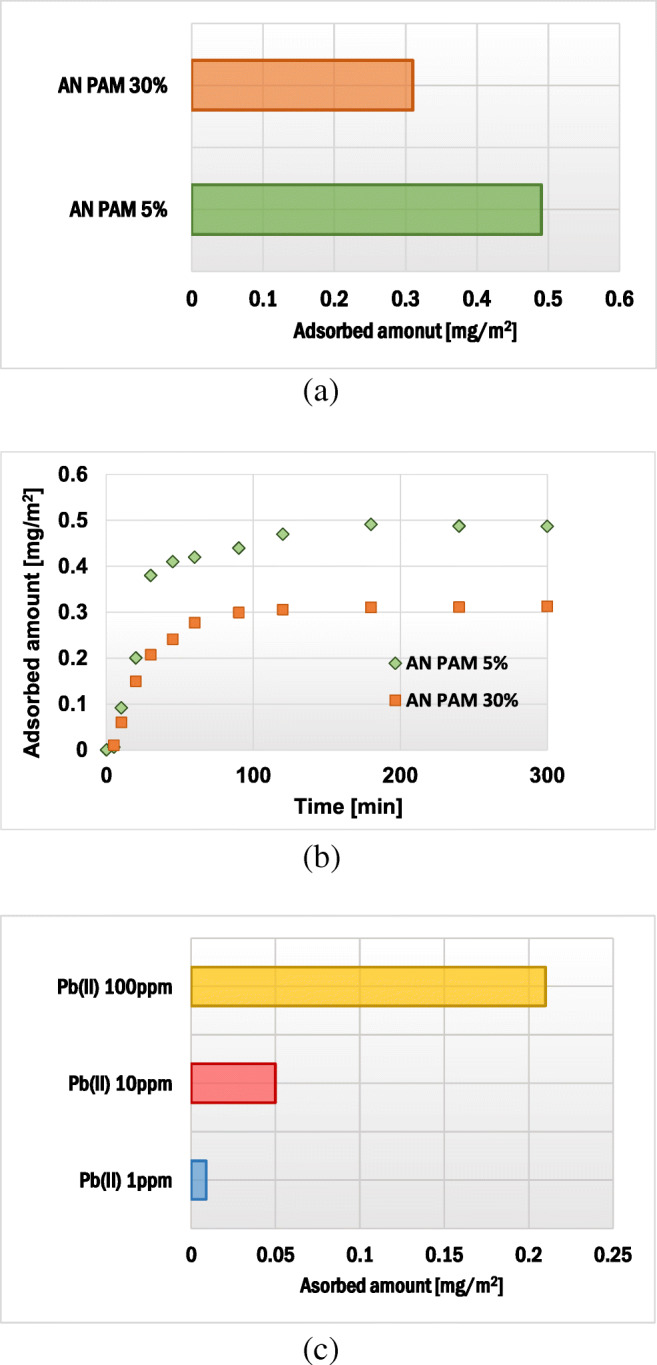
Adsorbed amount and adsorption kinetics of anionic polyacrylamide (**a**) as well as adsorbed amount of Pb(II) ions (**c**) on the montmorillonite surface

AN PAM and Pb(II) ions adsorbed amounts on the montmorillonite surface as well as AN PAM adsorption kinetics are presented in Fig. 2. Pb(II) adsorption kinetics on the selected solid are presented elsewhere [48]. Comparing the data obtained, larger AN PAM amount was absorbed in the case of AN PAM 5% – the polymer containing smaller number of carboxylic groups. This value of AN PAM 5% was 0.49 mg/m^2^, whereas of AN PAM 30% – 0.31 mg/m^2^. At the studied pH value (pH 5) the dissociation degrees of carboxylic groups present in AN PAM 5% and AN PAM 30% were 98 and 98.7%, respectively. Thus, almost all carboxylic groups of the polymer were ionized and the macromolecules were negatively charged. However, the number of ionizable groups in AN PAM 5% was so small that its macromolecules assumed more coiled conformation than the AN PAM 30% ones. As a result, more polymer chains of AN PAM 5% might be adsorbed on a unit solid surface. At pH 5 the montmorillonite surface was neutral and, due to this fact, anionic polyacrylamides were adsorbed on the clay mineral surface mainly through hydrogen bonds. These bonds were formed between the montmorillonite hydroxyl groups and the functional groups of polyacrylamide.

Anionic polyacrylamide adsorbed amount on kaolinite, another clay mineral, were also determined. At pH 5 it was equal to 0.32 mg/m^2^ for AN PAM 5% and 0.21 mg/m^2^ for AN PAM 30% [51]. Goethite and gibbsite were also examined as sorbents of AN PAM containing 40% of carboxylic groups. At pH 5 the polymer adsorbed amount on gibbsite equaled 1.4 mg/m^2^ [43], whereas on goethite – 0.79 mg/m^2^ [44]. All above values were obtained for initial AN PAM concentrations equal to 100 ppm.

The adsorbed amounts of lead(II) ions on montmorillonite for its initial concentrations equal to 1, 10 and 100 ppm were 0.009, 0.05 and 0.21 mg/m^2^, respectively. This accumulation was based on two mechanisms: (1) the electrostatic attraction between surface hydroxyl groups of montmorillonite and Pb^2+^ ions as well as (2) the ion-exchange phenomenon. The cations such as Mg^2+^ or Ca^2+^ (so-called interlayered cations), present in the structure of 2:1 clay mineral (between the silica and alumina sheets), underwent exchange with Pb(II) ions [52, 53].

The Pb(II) adsorbed amount on kaolinite, for the initial Pb(II) concentration 10 ppm, was equal to 0.09 mg/m^2^ [51]. Kennedy Oubagaranadin and Murthy [54] calculated the maximum monolayer adsorption capacity of montmorillonite-illite clay – it was equal to 52 mg/g. Janssen et al. [55] found that montmorillonite-Al hydroxide polymer systems can bind even 1.35 mol kg^−1^ clay.

### Dependence of AN PAM adsorbed amount on the adsorbate addition sequence and Pb(II) ions concentration

**Fig. 3:**
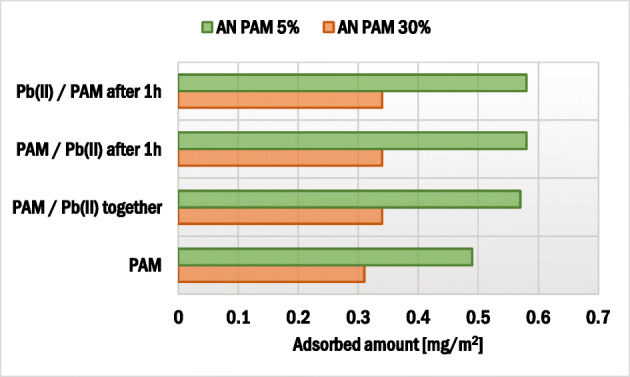
Influence of the sequence of AN PAM and Pb(II) addition on the anionic polyacrylamide adsorbed amount on the montmorillonite surface

**Fig. 4:**
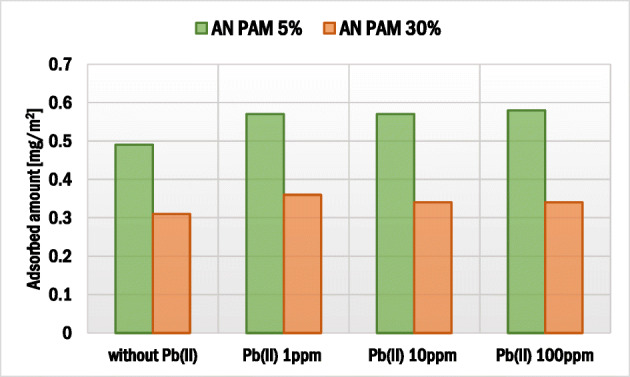
Influence of concentration of Pb(II) ions on the AN PAM adsorbed amount on the montmorillonite surface; both adsorbates were added together

The effects of adsorbate addition sequence as well as Pb(II) ion concentration on the polyacrylamide adsorbed amount on the montmorillonite surface are presented in Figs. 3 and 4. The AN PAM and Pb(II) addition sequence had a minimal effect on the AN PAM 5% and AN PAM 30% adsorbed amounts on the montmorillonite surface. In both examined situations, i.e. when the adsorbates were added to the system simultaneously or in intervals of time, the AN PAM adsorbed amount was the same. Such phenomenon was observed for all tested concentrations of Pb(II) ions, i.e. 1, 10 and 100 ppm. Due to large size of AN PAM macromolecules, they cannot freely penetrate the montmorillonite pores and internal structure. On the other hand, lead(II) ions are small in size and thus may be adsorbed within pores of the solid (the mean size of pores is 5.9 nm). What is more, due to the intercalation phenomenon, heavy metal ions can be located in the interlayer spaces in the 2:1 clay mineral. It is also worth mentioning that, due to the electrostatic attraction between the negatively charged carboxylic groups in the PAM macromolecules and the positive lead(II) ions, formation of polymer-metal complexes may occur. As a result, the increase in the AN PAM adsorbed amount in the presence of Pb(II) ions was observed. Nevertheless, the noted change was not very large, which suggested that the metal-polymer complexes were of intra-molecular character (several lead(II) ions were bound within one PAM macromolecule). Formation of inter-molecular complexes was also possible but the obtained adsorption results indicated that they were of marginal importance.

### Effects of adsorbates addition sequence and AN PAM presence on the Pb(II) adsorbed amount

**Fig. 5:**
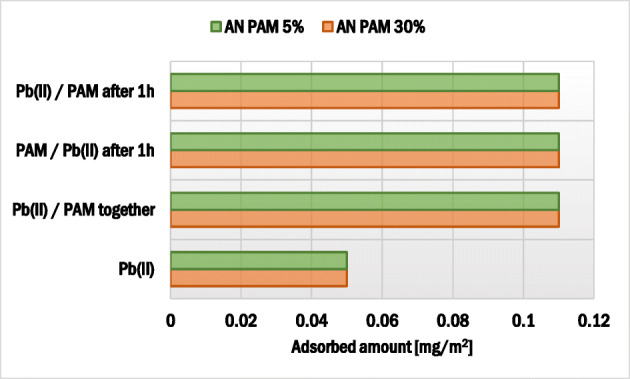
Influence of the sequence of PAM and Pb(II) addition on the lead(II) ions adsorbed amount on the montmorillonite surface

**Fig. 6:**
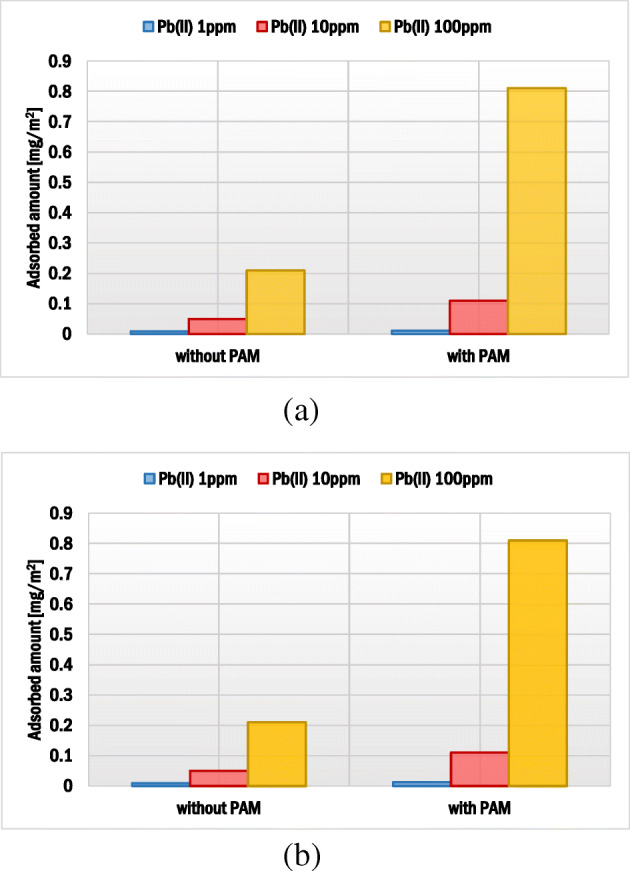
Influence of concentration of Pb(II) ions on the lead(II) ions adsorbed amount in the presence of: (**a**) AN PAM 5% and (**b**) AN PAM 30% on the montmorillonite surface; both adsorbates were added together

The impacts of adsorbate addition sequence and AN PAM concentration on lead(II) adsorbed amount on montmorillonite are presented in Figs. 5 and 6. The sequence of AN PAM and Pb(II) ions addition did not affect the lead adsorption significantly. However, the addition of anionic polyacrylamide had a clear impact on the heavy metal ions bounding. In the system with initial Pb(II) concentration equal to 1 ppm, a slight increase in the heavy metal adsorbed amount in the presence of anionic polyacrylamide was observed. In turn, when the initial Pb(II) concentration was 100 ppm, the Pb(II) adsorbed amount was four times larger compared to the system without AN PAM. Thus, it can be stated that, due to the formation of mainly intra-molecular complexes between lead(II) ions and anionic groups of polyacrylamide, a significant increase in Pb(II) ions adsorption occurred. Both AN PAM 5% and AN PAM 30% captured lead(II) from the solution effectively and the enhanced heavy metal accumulation on the solid was observed.

In previous study, the impact of anionic polyacrylamide on the Pb(II) immobilization on kaolinite was examined. In that case, the selected polymer enhanced the heavy metal accumulation on the solid [51]. Szewczuk-Karpisz et al. [24] found that anionic polyacrylamide favoured Cu(II) adsorption on the hay-based activated biochar. In turn, Aziz et al. [56] claimed that humic acids decreased the Pb(II) adsorbed amount on montmorillonite.

### Electrokinetic properties of montmorillonite particles without and with AN PAM and Pb(II) ions

**Fig. 7:**
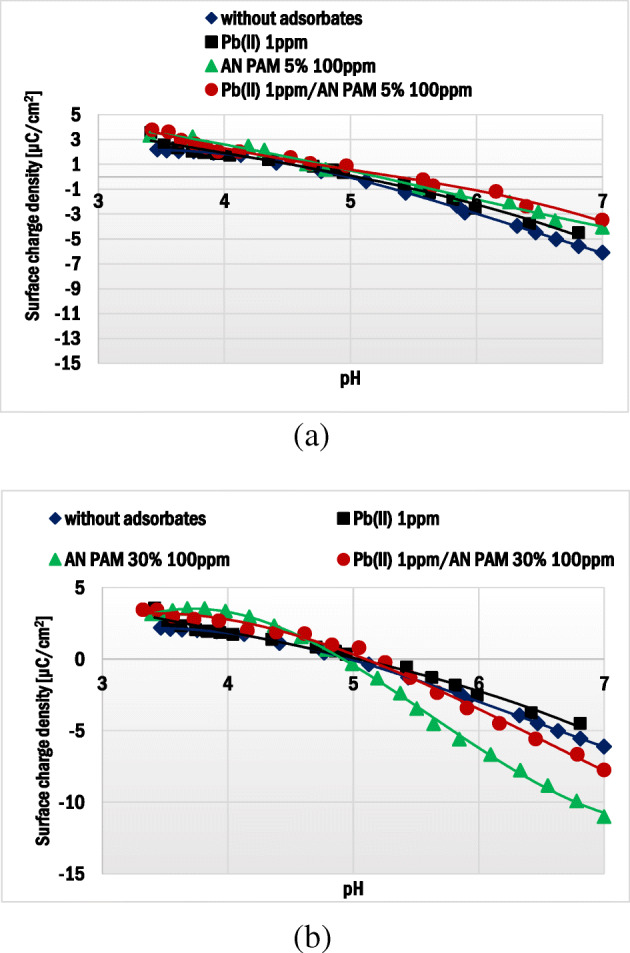
Surface charge density of montmorillonite without and with Pb(II) in the presence of: (**a**) AN PAM 5% and (**b**) AN PAM 30% as a function of solution pH

The results of potentiometric titration and zeta potential measurements showed electrokinetic properties of the montmorillonite suspension without and with AN PAM and/or Pb(II) ions. The montmorillonite surface charge density (σ_0_) in the examined systems is presented in Fig. 7. The pH_pzc_ (point of zero charge) of the mineral was about 5.02. At this pH value the concentrations of positive and negative groups were the same and the total surface charge was equal to 0. The anionic polyacrylamide addition changed the surface density of the solid. In the presence of AN PAM 30%, at pH < 5 a slight increase in the σ_0_ values was observed, whereas at pH > 5 the solid surface charge became more negative. These changes were induced by the –COO^−^ groups of the PAM macromolecules. Due to almost complete dissociation, these groups were very numerous. Most of them were present in the non-adsorbed AN PAM segments, i.e. in the ‘tail’ and ‘loop’ structures of adsorbed macromolecules. Due to higher content of negatively charged carboxylic groups in AN PAM 30% (in comparison to the AN PAM 5% ones), this polymer contributed to more evident changes in the montmorillonite surface charge density.

The presence of Pb(II) ions in the studied systems caused insignificant changes σ_0_ within the whole examined pH range (Fig. 7). According to the site-binding model [57], lead(II) ions can interact with the surface hydroxyl groups of montmorillonite based on the reactions:

4 $$ \equiv SOH+{Ct}^{2+}\leftrightarrow \equiv {SO}^{-}{Ct}^{2+}+{H}^{+} $$

5 $$ 2\left(\equiv SO H\right)+{Ct}^{2+}\leftrightarrow {\left(\equiv SO\right)}_2{Ct}^{2+}+2{H}^{+} $$

6 $$ \equiv SOH+{Ct}^{2+}+{H}_2O\leftrightarrow \equiv {SO}^{-}{ Ct OH}^{+}+2{H}^{+} $$

The adsorption of lead(II) ions may contribute to the creation of negative sites on the solid surface. Nevertheless, this effect was not clear in the examined systems due to low concentration of heavy metal ions (1 ppm).

In the systems containing AN PAM and lead(II) ions at the same time, insignificant changes in the solid surface charge density were observed. Within the AN PAM-Pb(II) complexes, many negatively charged carboxylic groups in the PAM chains are involved in lead(II) ions binding. As a result, a small number of polymer functional groups may interact with the solid surface (especially in the case of AN PAM 5%).

**Fig. 8:**
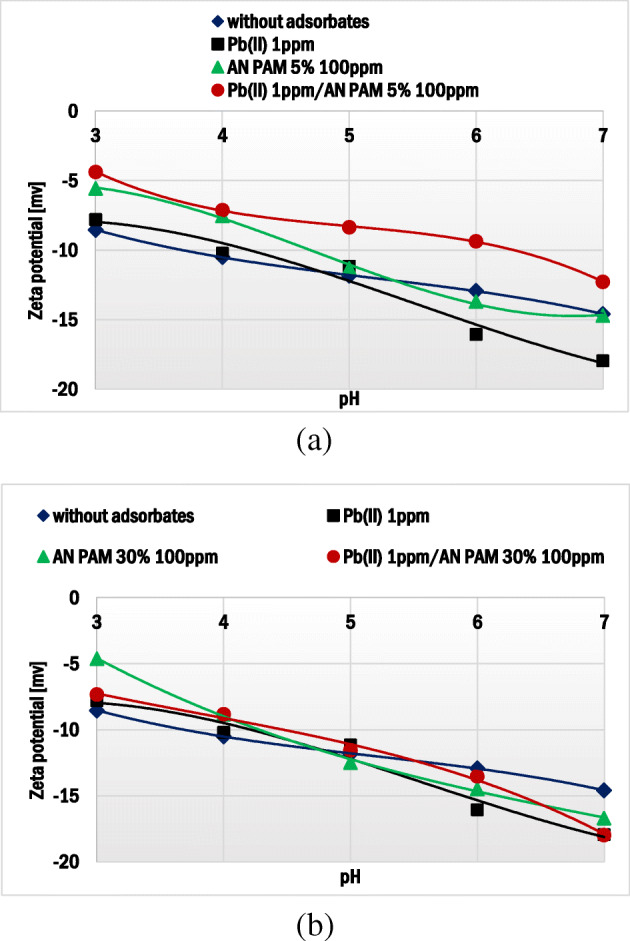
Zeta potential of montmorillonite particles without and with Pb(II) in the presence of: (**a**) AN PAM 5% and (**b**) AN PAM 30% as a function of solution pH

Zeta potential of montmorillonite without and with AN PAM and/or Pb(II) ions is presented in Fig. 8. The iep parameter (isoelectric point) of the studied mineral was lower than 3, which was manifested by negative values of its electrokinetic potential in the whole examined pH range. The addition AN PAM 5% or AN PAM 30% caused small changes in the zeta potential of the mineral particles. In the mixed systems or those containing only lead(II) ions, the observed zeta potential changes were also slight. These phenomena were caused by two overlapping effects. The first was associated with the slipping plane offset by the adsorbed PAM macromolecules which was mainly responsible for the reduction of montmorillonite zeta potential values [58]. The second effect was based on the ion-exchange between the cations: interlayered (Mg^2+^, Ca^2+^) and heavy metal (Pb^2+^), which made zeta potential values higher. The above effects were simultaneous and, as a result, the changes in the ionic composition of the montmorillonite slipping plane and diffusion layer were small. Slightly more significant changes noted for the systems containing AN PAM 5% (compared to AN PAM 30%) were probably associated with different conformations of the polymer chains and AN PAM-Pb(II) complexes in the adsorption layer.

### Influence of AN PAM and Pb(II) on the montmorillonite suspension stability

**Fig. 9:**
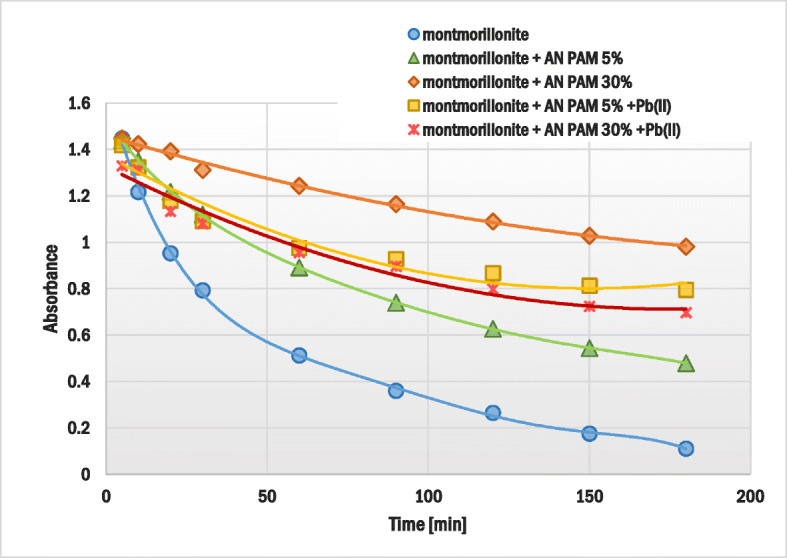
Changes in absorbance over time for the montmorillonite suspension, without and with the anionic polyacrylamide/Pb(II) ions

The addition of anionic polyacrylamide with or without Pb(II) ions changed the montmorillonite suspension stability significantly. The results of the sedimentation study are presented in Fig. 9. The system containing anionic polymer with or without heavy metal ions showed a higher absorbance (even after 180 min from the measurement start) in comparison to the system without the adsorbates. Thus, AN PAM increased stability of the montmorillonite aqueous suspension. This phenomenon resulted probably from steric and electrostatic repulsion between the solid particles covered with polymeric layers (electrosteric effect). The largest impact was observed for AN PAM 30%, which was mainly associated with high content of carboxylic groups in its macromolecules. At pH 5 these moieties were dissociated and, due to this fact, the electrostatic forces occurring between the montmorillonite particles with adsorbed polymer were very strong. The AN PAM 5% impact on the system stability was not as significant as that of AN PAM 30% because in this case the number of groups arranged in electrostatic interactions was lower.

## Conclusions

Based on the obtained results of adsorption and electrokinetic measurements there can be drawn the following conclusions:

1. At pH 5, due to more coiled structure of polymer macromolecules, higher adsorbed amount was observed for AN PAM 5%. For the initial polymer concentration 100 ppm, the AN PAM 5% adsorbed amount equaled 0.49 mg/m^2^, whereas for AN PAM 30% – 0.31 mg/m^2^.
2. The lead(II) ions caused a slight increase in the AN PAM adsorbed amount on the montmorillonite surface due to the formation of metal-polymer complexes mainly of intra-molecular character. In the Pb(II) presence, the AN PAM 5% adsorbed amount was 0.57 mg/m^2^, whereas the AN PAM 30% one – 0.36 mg/m^2^.
3. Anionic polyacrylamide contributed to stronger Pb(II) ions accumulation on montmorillonite at pH 5. This was mainly caused by electrostatic attraction between the positively charged lead(II) ions and the negatively charged –COOH groups located in the polymer chains leading to the effective complexation process. For the initial Pb(II) concenctration 10 ppm, its adsorbed amount without the polymer was 0.05 mg/m^2^, whereas in the AN PAM 5% or AN PAM 30% presence – 0.11 mg/m^2^.
4. Sequence of individual adsorbates addition had practically no effect on anionic polyacrylamide and lead(II) ions adsorbed amounts on the mineral surface.
5. The optimum conditions for lead(II) and anionic polyacrylamide adsorption on montmorillonite were as follows: 25 °C, solid weight equal to 0.003 g, 24 h.
6. The addition of anionic polyacrylamide and lead(II) ions affects montmorillonite electrokinetic and stability properties.

## References

[1] Kowalski D, Wróbel M, Rybak J (2018). Ecotoxicological studies on the impact of copper smelter “Legnica” on living organisms. Ecol Eng.

[2] Piekut A, Krzysztofik L, Gut K (2018). Narażenie mieszkańców Zabrza na metale ciężkie emitowane z hałd poprzemysłowych. Ecol Eng.

[3] Bai L, Wang Y, Zhou Y, Liu L, Yan Z, Li F (2015). Research on the process-based risk evaluation method of groundwater pollution for contaminated site. Water Sci Technol Water Supply.

[4] Ouali N, Belabed BE, Zeghdoudi F, Rachedi M (2018). Assessment of metallic contamination in sediment and mullet fish (Mugil cephalus Linnaeus, 1758) tissues from the east Algerian coast. J Water Land Develop.

[5] McGrath SP, Cegarra J (1992). Chemical extractability of heavy metals during and after long term applications of sewage sludge to soil. Eur J Soil Sci.

[6] Olayiwola HA, Abudalawal L, Adewuyi GK, Azeez MO (2017). Heavy metal contents in soil and plants at dumpsites: a case study of Awotan and Ajakanga dumpsite Ibadan, Oyo state. Nigeria J Environ Earth Sci.

[7] Kumpiene J, Lagerkvist A, Maurice C (2008). Stabilization of as, Cr, cu, Pb and Zn in soil using amendments – a review. Waste Manag.

[8] Kuziemska B, Kalembasa S (2009). Influence of soil contamination with nickel and liming on lead and manganese contents in red clover biomass. Arch Environ Prot.

[9] Rieuwerts JS, Thornton I, Farago ME, Ashmore MR (1998). Factors influencing metal bioavailability in soils: preliminary investigations for the development of a critical loads approach for metals. Chem Spec Bioavailab.

[10] Macioszczyk A, Dobrzyński D (2002). Hydrochemia – strefy aktywnej wymiany wód podziemnych.

[11] Hermanowicz W, Dojlido J, Dożańska W, Koziorowski B, Zerbe J (1999). Fizyczno-chemiczne badanie wody i ścieków.

[12] Szymański K (2009). Związki ołowiu i chromu w środowisku naturalnym i odpadach. Rocznik Ochrona Środowiska.

[13] Gouder de Beauregard AC, Mahy G (2002). Phytoremediation of heavy metals: the role of macrophytes in stormwater basin. Ecohydrol Hydrobiol.

[14] Melo MR, Flores NR, Murrieta SV, Tovar AR, Zúñiga AG, Mendoza AP, Pérez NO, Dorantes AR, Hernández OF (2011). Comparative plant growth promoting traits and distribution of rhizobacteria associated with heavy metals in contaminated soils. Int J Environ Sci Tech.

[15] Bień JB (2007). Osady ściekowe: teoria i praktyka.

[16] Sas-Nowosielska A. Fitotechnologie w remediacji terenów zanieczyszczonych przez przemysł cynkowo-ołowiowy. Monografia nr 189, Wyd. Politechniki Częstochowskiej, Częstochowa; 2009.

[17] Chaney RL, Malik M, Li YM, Angle JS (1997). Phytoremediation of soil metals. Curr Opin Biotechnol.

[18] Pueyo M, Lopez-Sanchez JF, Rauret G (2004). Assessment of CaCl_2_, NaNO_3_, and NH_4_NO_3_ extraction procedures for the study of cd, cu, Pb and Zn extractability in contaminated soils. Anal Chim Acta.

[19] Jin CW, Zheng SJ, He YF, Zhou GD, Zhou ZX (2005). Lead contamination in tea garden soils and factors affecting its bioavailability. Chemosphere.

[20] Kabata-Pendias A, Mukherjee AB (2007). Trace elements from soil to human.

[21] Adamus ML, Zhao FJ, McGrath SP, Nicholson FA, Chambers BJ (2004). Heavy metals in the environment. Predicting cadmium concentrations in wheat and barley grain using soil properties. J Environ Qual.

[22] Mansoorian HJ, Mahvi AH, Jafari AJ (2014). Removal of lead and zinc from battery industry wastewater using electrocoagulation process: influence of direct and alternating current by using iron and stainless steel rod electrodes. Sep Purif Technol.

[23] Adamczuk A, Kołodyńska D (2017). Utilization of fly ashes from the coal burning processes to produce effective low-cost sorbents. Energ Fuel.

[24] Szewczuk-Karpisz K, Nowicki P, Sokołowska Z, Pietrzak R (2020). Hay-based activated biochars obtained using two different heating methods as effective low-cost sorbents: solid surface characteristics, adsorptive properties and aggregation in the mixed cu(II)/PAM system. Chemosphere..

[25] Wiśniewska M, Nowicki P (2019). Simultaneous removal of lead(II) ions and poly(acrylic acid) macromolecules from liquid phase using of biocarbons obtained from corncob and peanut shell precursors. J Mol Liq.

[26] Kalhor MM, Rafati AA, Rafati L, Rafati AA (2018). Synthesis, characterization and adsorption studies of amino functionalized silica nano hollow sphere as an efficient adsorbent for removal of imidacloprid pesticide. J Mol Liq.

[27] Kamaranifar M, Khodadadi M, Samiei V, Dehdashti B, Sepehr MN, Rafati L, Nasseh N (2018). Comparison the as a low cost adsorbent from aqueous solutions: isotherm and kinetic study. J Mol Liq.

[28] Rafati L, Ehrampoush MH, Rafati AA, Mokharti M, Mahvi AH (2018). Removal of ibuprofen from aqueous solution by functionalized strong nano-clay composite adsorbent: kinetic and equilibrium isotherm studies. Int J Env Sci Technol..

[29] Rafati L, Ehrampoush MH, Rafati AA, Mokharti M, Mahvi AH (2016). Modeling of adsorption kinetic and equilibrium isotherms of naproxen onto functionalized nano-clay composite adsorbent. J Mol Liq.

[30] Rafati L, Nabizadeh R, Mahvi AH, Dehghani MH (2012). Removal of phosphate from aqueous solutions by iron nano-particle resin Lewatit (FO36). Korean J Chem Eng.

[31] Rafati L, Ehrampoush MH, Rafati AA, Mokharti M, Mahvi A (2018). Nanocomposite adsorbent based on β-cyclodextrin-PVP-clay for the removal of naproxen from aqueous solution: fixed-bed column and modeling studies. Desalin Water Treat.

[32] Henriksen K, Berthelsen L, Maltzen R (1998). Separation of liquid pig manure by flocculation and ion exchange part 1: laboratory experiments. Res Agr Eng.

[33] Gui ZL, Qian JW, Li XK, Zheng BQ, An QF (2010). Study on chain deformation of polyacrylamides in solutions and its flocculation performance during the flocculation process. J Appl Polym Sci.

[34] Ma J, Shi J, Ding H, Zhu G, Fu K, Fu X (2017). Synthesis of cationic polyacrylamide by low-pressure UV initiation for turbidity water flocculation. Chem Eng J.

[35] Rasteiro MG, Garcia FAP, Ferreira PJ, Antunes E, Hunkeler D, Wandrey C (2010). Flocculation by cationic polyelectrolytes: relating efficiency with polyelectrolyte characteristics. J Appl Polym Sci.

[36] Mamedov AI, Huang C, Aliev FA, Levy GJ (2016). Aggregate stability and water retention near saturation characteristics as affected by soil texture, aggregate size and polyacrylamide application. Land Degrad Dev.

[37] Krishnamoorthi S, Adhikary P, Mal D, Singh RP (2010). Novel polymeric flocculants based on polyacrylamide grafted dextran in kaolin suspension. J Appl Polym Sci.

[38] Liang XQ, Liu ZW, Liu J, Chen LL, Tian GM (2017). Soil colloidal P release potentials under various polyacrylamide addition levels. Land Degrad Dev.

[39] Li Y, Shao M, Horton R (2011). Effect of polyacrylamide applications on soil hydraulic characteristics and sediment yield of sloping land. Procedia Environ Sci.

[40] Yang X, Chen K, Zhang Y, Liu H, Chen W, Yao J (2017). Polyacrylamide grafted cellulose as an eco-friendly flocculant: efficient removal of organic dye from aqueous solution. Fibers Polymer.

[41] Sepaskhah AR, Mahdi-Hosseinabadi Z (2008). Effect of polyacrylamide on the erodibility factor of a loam soil. Biosyst Eng.

[42] Hennecke D, Bauer A, Herrchen M, Wischerhoff E, Gores F (2018). Cationic polyacrylamide copolymers (PAMs): environmental half life determination in sludge-treated soil. Environ Sci Eur.

[43] Szewczuk-Karpisz K, Krasucka P, Boguta P, Skic K, Sokołowska Z, Fijałkowska G, Wiśniewska M (2018). Electrical double layer at the gibbsite/anionic polyacrylamide/supporting electrolyte interface – adsorption, spectroscopy and electrokinetic studies. J Mol Liq.

[44] Szewczuk-Karpisz K, Krasucka P, Boguta P, Skic K, Sokołowska Z, Fijałkowska G, Wiśniewska M (2019). Anionic polyacrylamide efficiency in goethite removal from aqueous solutions: goethite suspension destabilization by PAM. J Environ Sci Technol.

[45] Minczewski J, Marczenko Z (1965). Analytical chemistry.

[46] Crummett WB, Hummel RA (1963). The determination of traces of polyacrylamides in water. J Am Water Works Assoc.

[47] Dagnall RM, West TS, Young P (1965). Determination of lead with 4-(2-pyridylazo)-resorcinol—II: application to steel, brass and bronze. Talanta.

[48] Fijałkowska G, Szewczuk-Karpisz K, Wiśniewska M (2019). Chromium(VI) and lead(II) accumulation at the montmorillonite/aqueous solution interface in the presence of polyacrylamide containing quaternary amine groups. J Mol Liq.

[49] Gun'ko VM, Mikhailova IV, Zarko VI, Gerashchenko II, Guzenko NV, Janusz W, Leboda R, Chibowski S (2003). Study of interaction of proteins with fumed silica in aqueous suspensions by adsorption and photon correlation spectroscopy methods. J Colloid Interf Sci.

[50] Hunter RJ (1981). Zeta potential in colloid science.

[51] Fijałkowska G, Szewczuk-Karpisz K, Wiśniewska M (2020). Anionic polyacrylamide as a substance strengthening the Pb(II) immobilization on the kaolinite surface. Int J Env Sci Technol.

[52] Temuujin J, Okada K, MacKenzie KJD (2003). Preparation of porous silica from vermiculite by selective leaching. Appl Clay Sci.

[53] Hashem FS, Amin MS, El-Gamal SMA (2015). Chemical activation of vermiculite to produce highly efficient material for Pb^2+^ and Cd^2+^ removal. Appl Clay Sci.

[54] Kennedy Oubagaranadin JU, Murthy ZVP (2009). Adsorption of divalent lead on montmorillonite-illite type of clay. Ind Eng Chem Res.

[55] Janssen RPT, Bruggenwert MGM, Van Dijk G, Van Riemsdijk WH (2007). Lead ion adsorption on montmorillonite-Al hydroxide polymer systems. Eur J Soil Sci.

[56] Aziz I, Sirajuddin M, Khan MH, Naseem S, Trimizi SA, Khan R (2015). Investigation of adsorption of lead(II) onto montmorillonite clay modified by humic acid. J Chem Soc Pakistan.

[57] Wiśniewska M, Chibowski S, Urban T (2017). Comparison of adsorption affinity of ionic polyacrylamide for the surfaces of selected metal oxides. Ads Sci Technol.

[58] Chibowski S, Wiśniewska M (2001). Study of the adsorption mechanism and the structure of adsorbed layers of polyelectrolyte at metal oxide-solution interface. Ads Sci Tech.

